# Receptor‐Dependent and ‐Independent Effects of Hemin on Platelet Plasma Membrane Disintegration

**DOI:** 10.1096/fj.202503706R

**Published:** 2026-01-12

**Authors:** Zoi Laspa, Anne‐Katrin Rohlfing, Ravi Hochuli, Pamela Weronika Sowa, Tatsiana Castor, Meinrad Paul Gawaz

**Affiliations:** ^1^ Department of Cardiology and Angiology University Hospital Tübingen, University Tübingen Tübingen Germany

**Keywords:** deferoxamine, ferroptosis, hemin, ITAM‐signaling, platelets, recombinant soluble Fc‐GPVI

## Abstract

Microhemorrhages are an underestimated aspect in the pathophysiology of vulnerable plaques and aneurysms. Erythrocyte liberation within hemorrhages leads to extracellular hemoglobin accumulation and iron‐containing hemin generation. Hemin induces platelet activation, thrombosis, and ferroptosis‐mediated destruction of platelet membranes through GPVI/CLEC‐2 signaling. Hemin‐toxicity results in destruction of platelet membranes, which is caused by ferroptosis, a non‐apoptotic cell death. Antiplatelet drugs have limited effect on hemin‐induced activation. We evaluated the effect of hemin on platelet function using light transmission aggregometry and multipanel flow cytometry. We found that P2Y_12_ and COX‐1 inhibition attenuates hemin‐induced aggregation only at low hemin concentrations (3.1/6.25 μM), whereas at higher concentrations (12.5/25 μM) no substantial inhibition was found. High hemin concentrations enhance phosphatidylserine exposure, procoagulant and microvesicle formation as well as ferroptosis, which was not attenuated in the presence of Src‐inhibitors, indicating that membrane‐disintegration is not primarily mediated via GPVI/CLEC‐2 receptor‐dependent ITAM‐signaling. In contrast, iron chelation by deferoxamine significantly reduced microvesicle and ROS generation, loss of mitochondrial membrane potential and lipid peroxidation. Soluble recombinant Fc‐GPVI scavenging of hemin protects against hemin‐induced platelet activation, plasma membrane disintegration and microvesicle formation. High hemin concentrations lead to plasma membrane disintegration and ferroptosis, inhibited by iron chelation and hemin scavenging via soluble Fc‐GPVI, but not by GPVI/CLEC‐2 receptor‐mediated ITAM signaling. We speculate that iron overload enables receptor‐independent ferroptosis induction by hemin and may represent a therapeutic target to prevent platelet‐driven thrombosis in microhemorrhages.

## Graphical abstract

Microhemorrhages lead to the release of erythrocytes, which are then degraded and lysed. These hemolyses result in the liberation of free iron‐containing hemin. Hemin induces platelet activation and ferroptosis. Inhibition of GPVI/CLEC‐2 receptor‐mediated ITAM‐signaling inhibits hemin‐induced platelet activation (low hemin concentrations). Deferoxamine, an iron‐chelator, and hemin scavenging by soluble Fc‐GPVI receptor prevent platelet ferroptosis and plasma membrane destruction (high hemin concentrations).

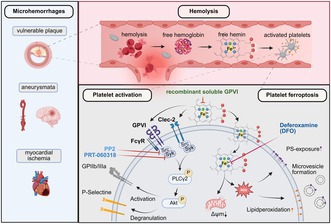

## 1 Introduction

At sites of tissue injury, intramural hemorrhages frequently occur in the process of various cardiovascular diseases including acute myocardial infarction, acute aortic syndrome, and vulnerable atherosclerotic plaques [1, 2, 3]. Intramyocardial microbleeding during reperfusion after acute myocardial infarction promotes delayed tissue healing and adverse cardiac remodeling [4]. Further, intraplaque microhemorrhages contribute to plaque instability and progression of coronary atheroma as well as in the progression of aneurysm of larger arteries [1, 5].

Microhemorrhages lead to the extravascular accumulation of erythrocytes, which undergo lysis, thereby releasing free hemoglobin. Free hemoglobin is degraded, which results in the liberation of the free ferrous iron‐containing heme and its oxidized ferric‐iron containing metabolite, hemin [6]. Within the hematoma of intracerebral hemorrhages, free hemin accumulation can reach up to 3 mM, whereas the surrounding perihematomal tissue can still contain up to 200 μM of free hemin [2]. Plasma hemin concentrations in trauma patients are increased nearly 10‐fold compared to healthy controls [7]. Previously, the iron‐containing porphyrin hemin has been shown to promote platelet activation and aggregation, which may contribute to limiting ongoing bleeding at sites of tissue injury [8, 9, 10]. Besides its role in hemostasis, the interaction of hemin with platelets promotes thrombus formation and thrombo‐inflammation, which in turn may augment disease progression and limit tissue or vascular regeneration [4, 11, 12]. Because of the excessive accumulation of hemin, the endogenous scavenger systems with haptoglobin, hemopexin, and albumin may become overwhelmed, and hemin reveals its full proinflammatory and prothrombotic potential [13].

Recently, it was shown that hemin induces platelet activation and aggregation via the collagen receptor glycoprotein VI (GPVI) and the C‐type lectin‐like receptor 2 (CLEC‐2) [9, 10]. Although other receptors such as Toll‐like receptor 4 (TLR‐4) have been shown to bind hemin, the GPVI/CLEC‐2 complex has been postulated to be the primary hemin‐responsive surface receptor expressed on the platelet plasma membrane [10, 14]. Interaction of hemin with GPVI/CLEC‐2 activates the immunoreceptor tyrosine‐based activation motif (ITAM) signaling pathway, leading to the phosphorylation of Syk kinase, PLCγ2, and protein kinase B (Akt) [9, 10, 15]. Consequently, the fibrinogen receptor (αIIbβ3, GPIIb‐IIIa) becomes activated and degranulation of α‐, δ‐granules, and lysosomes occurs [16, 17, 18]. Further, hemin has been shown to induce ferroptosis in platelets in a concentration‐dependent manner (Figure S1A–D), a form of cell death characterized by the loss of mitochondrial potential (ΔΨm), increased reactive oxygen species generation (ROS), lipid peroxidation, exposure of phosphatidylserine (PS), and disintegration of the plasma membrane, which can be regulated by the subtilisin‐like proprotein convertase furin [8, 19, 20, 21]. Even though hemin leads to the depolymerization of the mitochondrial membrane, PS exposure, and microvesicle formation, it does not activate caspase‐3 and does not trigger the release of cytochrome C in the cytosol, which are key players in the apoptotic process [8, 20].

Hemin‐dependent platelet activation can only be partially inhibited by conventional platelet antagonists such as cyclooxygenase 1 (COX‐1) inhibitors and P2Y_12_ antagonists [10, 15, 22]. Thus, conventional anti‐platelet therapies may fail to prevent hemin‐induced thrombotic events. Recently, hemin‐induced platelet activation and degranulation have been shown to be modulated by elevated cyclic guanosine monophosphate (cGMP) levels, atypical chemokine receptor 3 (ACKR3) agonism and inhibition of the proprotein convertase furin [8, 15, 18, 22].

The effect of hemin on platelet function can only be partially explained by specific receptor‐dependent signaling events, since at high concentrations (> 12.5 μM) hemin induces disintegration of the plasma membrane and ferroptosis [8].

The aim of the present study is to further elucidate the effect of hemin on platelet activation and plasma membrane disintegration with a focus on high hemin concentrations (12.5 and 25 μM) to distinguish between specific receptor‐dependent and ‐independent signaling events and to disclose potential therapeutic aspects.

## 2 Materials and Methods

### 2.1 Chemicals and Antibodies

Monoclonal anti‐human PAC‐1::FITC antibody was obtained from BD Bioscience (Franklin Lakes, NJ, USA). Anti‐human CD62P::PE antibody and CD42b::PE were obtained from Beckman Coulter (Brea, CA, USA). Annexin A5::APC and Annexin A5::FITC were received from ImmunoTools (Friesoythe, Germany). BODIPY 581/591 C11 and Tetramethylrhodamin ethylester (TMRE) were obtained from Invitrogen (Carlsbad, CA, USA). Hemin, indometacin (COX‐1 inhibitor), PP2 (Src kinase inhibitor), deferoxamine mesylate salt (DFO), 2′, 7′‐dichlorfluorescein diacetate (H_2_DCFDA), fibrinogen from human plasma, hemin‐agarose, and ferric chloride were obtained from Sigma Aldrich (St. Louis, MO, USA). Cangrelor (KENGREXAL 50 mg) was obtained from Chiesi Farmaceutici (Parma, Italy). PRT‐060318 (Syk kinase inhibitor) was obtained from MedChemExpress (Monmouth Junction, New Jersey, USA). Anti‐PLCγ2, anti‐phospho‐PLCγ2 (Tyr759), anti‐Akt, anti‐phospho‐Akt (Ser473), and anti‐α‐tubulin antibodies were all obtained from Cell Signaling Technology (Danvers, Massachusetts, USA). Donkey anti‐rabbit IR‐Dye 680RD, donkey anti‐rabbit IR‐Dye 800CW, and donkey anti‐mouse IR‐Dye 800CW secondary antibodies were obtained from Li‐COR (Lincoln, NE, USA). μMACS Protein A MicroBeads were obtained from Miltenyi Biotec (Bergisch Gladbach, Germany). Ferroprotoporphyrin IX was obtained from LGC Standards (Teddington, England). Soluble Fc‐GPVI (Revacept), Fc‐control, Fab Y020347 (anti‐GPVI), and anti‐GPVI (1A5) were obtained from AdvanceCOR (Martinsried, Germany).

### 2.2 Isolation of Human Platelets

Blood samples were collected from healthy donors who provided written informed consent. The procedure was approved by the Ethics Committee of the Medical Faculty of the Eberhard Karls University and the University Hospital of Tübingen (ethics vote 238/2018B02). Platelet isolation was performed as previously described [23, 24]. In short, blood was collected into syringes pre‐filled with acid‐citrate‐dextrose (ACD) anticoagulant at a 1:5 ratio. The samples were centrifuged at 209 *g* for 20 min without brakes to obtain platelet‐rich plasma (PRP). The PRP was carefully transferred into Tyrode's buffer (pH 6.5), consisting of 137 mM NaCl, 2.8 mM KCl, 12 mM NaHCO_3_, 5 mM glucose and 10 mM HEPES. The second centrifugation was then performed at 836 *g* for 10 min with the brakes applied. Afterward, the supernatant was discarded and the platelet pellet resuspended in Tyrode's buffer adjusted to pH 7.4. Platelet concentration was measured using a conventional cell counter (Sysmex Coorporation, Kobe, Japan).

### 2.3 Flow Cytometry

For standard flow cytometry platelets were adjusted to a count of 1 × 10^6^ per sample in Dulbecco's phosphate buffered saline (0.5 mM MgCl_2_, 0.9 mM CaCl_2_). In general, Fc‐GPVI was preincubated with higher hemin concentrations in a defined volume. From this Fc‐GPVI‐hemin mixture, a specific volume was then taken to reach the final hemin concentrations in every assay. To ensure the final hemin concentrations, hemin was always present in excess. Fc‐GPVI or Fc‐control (1.5 mg/mL) was preincubated with indicating hemin concentrations for 15 min at room temperature and then added as a Fc‐GPVI or Fc‐control/hemin mixture to the platelets. Then, samples were stimulated at indicated concentrations with hemin for 30 min at room temperature in the presence of PAC‐1::FITC (1:20) and CD62P::PE (1:20) monoclonal antibodies. The reaction was stopped by the addition of 250 μL 0.5% paraformaldehyde solution (PFA). The mitochondrial potential and phosphatidylserine exposure were measured by incubating the platelets after stimulation with 10 μM of TMRE or Annexin A5::FITC for 15 min at room temperature. Here, after incubation, the samples were diluted with 250 μL of Dulbecco's phosphate buffered saline. Measurements were performed using a FACS Calibur (BD Biosciences, Franklin Lakes, New Jersey, USA).

Multicolor flow cytometry was performed with platelets adjusted to 1 × 10^6^ per sample in binding buffer (1×) [15]. Where indicated, platelets were preincubated for 15 min with 200 μM deferoxamine or 5 min with 20 μM PP2 at room temperature. After 30 min of incubation with hemin, the antibodies and dyes Annexin A5::APC (1:20), CD42b::PE (1:20), and PAC‐1::FITC (1:20) were added to stain the platelets for 15 min at room temperature. Afterwards, the samples were diluted with 300 μL of binding buffer (1×) and immediately measured by using a FACSLyric (BD Bioscience, San Jose, CA, USA) flow cytometer.

For analyzing the microvesicle formation, isolated human platelets were prepared as described under the standard flow cytometry protocol. Platelets were incubated with indicated concentrations of hemin or Fc‐GPVI or Fc‐control/hemin‐mixture for 30 min at room temperature. Then, the samples were fixed with 0.5% paraformaldehyde and FSC/SSC data were collected to 30 000 events by FACSLyric (BD Bioscience, San Jose, CA, USA). Additionally, a suspension of 1 μm beads (Invitrogen) was measured to identify the microvesicle under 1 μm. The microvesicle percentage represents the fraction of events smaller than 1 μm compared with the total platelet event count.

The raw data of all flow cytometer measurements were analyzed using FlowJo software (version 10.10.0 FlowJo CCL, Ashton, OR, USA).

### 2.4 Platelet Light Transmission Aggregometry

Light transmission aggregometry experiments were performed with washed platelets adjusted to 2 × 10^7^ per sample containing 2 mM Ca^2+^ and 100 μg/mL fibrinogen as previously described [25]. Where indicated, platelets were preincubated with 10 μM indometacin, 10 μM cangrelor, or the dual inhibition (10 μM indometacin + 10 μM cangrelor) for 15 min at 37°C. Aggregation was induced by hemin or the Fc‐GPVI/hemin‐mixture and measured using a light transmission aggregometer (CHRONO‐LOG Aggregometer 490, Chrono‐log Corporation, Havertown, PA, USA) over 5.30 min at 37° with continuous stirring at 1000 rpm.

### 2.5 Measurement of Reactive Oxygen Species (ROS)

Reactive oxygen species (ROS) generation induced by hemin was quantified using the fluorescence probe 2′, 7′‐dichlorfluorescein diacetate (H_2_DCFDA) and measured with a plate reader (GloMax‐Multi Detection System, Promega GmbH, Walldorf, Germany). Platelets were adjusted at a concentration of 1.5 × 10^6^/μL in a total volume of 50 μL. Platelets were preincubated with 5 μM H_2_DCFDA for 15 min at room temperature in the dark. Where indicated, platelets were pre‐treated with 200 μM deferoxamine (15 min at RT) or PP2 (5 min at RT). After preincubation, Tyrode's buffer (pH 7.4) supplemented with 1 mM CaCl_2_ was added to reach a final volume of 100 μL. Then, the platelets were stimulated with hemin for 30 min at room temperature and the fluorescence of H_2_DCFDA was measured to assess ROS levels.

### 2.6 Measurement of Lipid Peroxidation

Lipid peroxidation was detected by measuring the fluorescence of the lipid peroxidation sensor BODIPY‐C11 with a plate reader (Infinite 200 Pro, Tecan Trading AG, Switzerland). Platelet were adjusted at a concentration of 1 × 10^6^/μL in a total volume of 50 μL. Platelets were preincubated with 10 μM BODIPY‐C11 for 15 min at 37°C in the dark. Where indicated, platelets were pre‐treated with 200 μM deferoxamine (15 min at RT) or PP2 (5 min at RT). After preincubation, Tyrode's buffer (pH 7.4) supplemented with 1 mM CaCl_2_ was added to reach a final volume of 100 μL. Then, the platelets were stimulated with hemin for 30 min at room temperature and the fluorescence of BODIPY‐C11 (Ex/Em 580/600 nm) was measured to detect the lipid peroxidation.

### 2.7 Immunoblot Analysis

Isolated human platelets (2.5 × 10^8^ per sample) were activated with hemin for 10 min (detection of pPLCγ2) and 20 min (detection of pAkt) at room temperature. Where indicated, the platelets were preincubated with 20 μM PP2 for 5 min at room temperature prior to stimulation with hemin. Cells were lysed with RIPA lysis buffer (50 mM TRIS/HCl, 150 mM NaCl, 0.1% SDS, 1% Triton‐X 100, 0.5% Na‐Deoxycholate) with supplemented protease cocktail (1:100) and protease/phosphatase inhibitor (1:100) (Cell Signaling Technology, Danvers, Massachusetts, USA) and incubated for at least 10 min on ice. Platelet lysates were mixed with sodium dodecyl sulfate (SDS) containing loading buffer (Carl Roth GmbH + Co. KG, Karlsruhe, Germany) and separated by sodium dodecyl sulfate‐polyacrylamide gel electrophoresis (SDS‐PAGE) using either an 8% polyacrylamide gel for PLCγ2 detection or a 10% acrylamide gel for Akt detection. Proteins were transferred to PVDF membrane (Sigma Aldrich, St. Louis, MO, USA) using a wet blot technique for 65 min at 100 V. Membranes were blocked for 1 h at room temperature with 5% BSA in tween‐tris buffered saline (TTBS). After that, the membranes were incubated overnight at 4°C with primary antibodies (1:500 anti‐phospho‐PLCγ2; 1:500 anti‐phospho‐Akt). Following primary antibody incubation, membranes were labeled with appropriate IRDye‐conjugated secondary antibodies for 90 min at room temperature. Equal protein loading was confirmed by anti‐α‐tubulin staining (1:1000). After drying, membranes were scanned and analyzed by Li‐COR‐Odyssey System (LI‐COR Biotechnology GmbH, Bad Homburg, Germany).

### 2.8 Live‐Cell Imaging on Fibrinogen

Fibrinogen (100 μg/mL) coated coverslips were incubated overnight at 4°C. The next day, the coverslips were washed with Dulbecco's phosphate buffered solution (PBS) and blocked with 1% bovine serum (BSA) for 1 h at room temperature. Isolated platelets were adjusted to 20 000 platelets/μL with 1 mM CaCl_2_. If indicated platelets were preincubated for 15 min with 200 μM deferoxamine and then stimulated with 25 μM hemin, FeCl_3_ or ferroprotoporphyrin IX. Images were taken after 30 min of stimulation with a Nikon Eclipse Ti2 microscope (Nikon Instruments Europe BV, Amsterdam, The Netherlands). Five images of randomly selected areas were analyzed per each treatment from three different healthy donors by counting the number of morphological phenotypes.

### 2.9 Absorbance Measurement of Hemin (Hemin‐Soluble Fc‐GPVI Binding)

Superparamagnetic Protein A beads were used to capture preincubated Fc‐GPVI‐hemin complex or a corresponding FcIgG control. Fc‐GPVI (50 μg/mL, as recommended by the manufacturer for bead binding) or Fc‐IgG was preincubated with 25 μM hemin for 15 min at room temperature. Subsequently, the beads were added and the mixture was incubated for 3 h at 4°C. After incubation, the beads were collected using a magnet and the supernatant was removed. The remaining beads with bound Fc‐GPVI‐hemin or FcIgG were analyzed using a plate reader (Infinite 200 Pro, Tecan Trading AG, Switzerland) by measuring the absorbance of hemin at 405 nm.

### 2.10 Immunoprecipitation of Fc‐GPVI (Hemin‐Soluble Fc‐GPVI Binding)

Hemin‐agarose beads (100 μM) were preincubated with 1.5 mg/mL Fc‐GPVI, a FcIgG control or Dulbecco's phosphate buffered solution (PBS) for 3 h at 4°C. After incubation, the beads were washed five times with PBS and the Fc‐GPVI was eluted using 3× reducing SDS loading buffer containing dithiothreitol (DTT; 1:30). The eluate was applied to a 10% acrylamide gel, wet blotted for 1 h at 100 V and detected using a primary anti‐GPVI antibody (1.5 mg/mL; clone: 1A5).

### 2.11 Statistics and Graphical Presentation

Statistical analysis and graphical data presentation were performed using GraphPad Prism (Version 10.1.1; GraphPad software Inc., La Jolla, CA, USA, Version 10.1.1). For comparison between two sets of normally distributed data, a paired Student's *t*‐test was applied. One‐way ANOVA or mixed‐effects analysis was used for comparison involving more than two groups. Data are presented as mean ± standard deviation (SD). The graphical abstract was created with BioRender.

## 3 Results

### 3.1 Inhibition of P2Y_12_ and COX‐1 Attenuates Platelet Aggregation Stimulated by Low but Not High Hemin Concentrations

**FIGURE 1:**
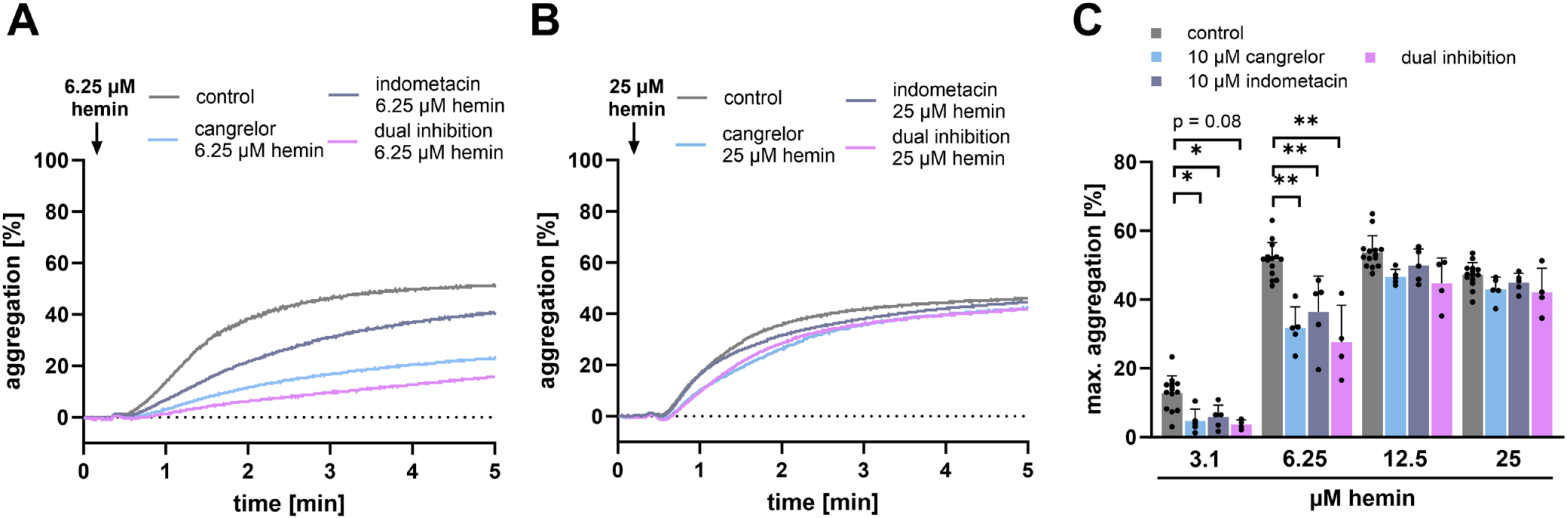
P2Y_12_ and COX1 blockers attenuate platelet aggregation stimulated by low hemin concentrations but not high hemin concentrations. (A–C) Light transmission aggregometry measurements. Representative traces of (A) 6.25 μM and (B) 25 μM hemin‐induced aggregation with 15 min pre‐treatment of 10 μM indometacin, 10 μM cangrelor or the dual inhibition (10 μM indometacin + 10 μM cangrelor). (C) Hemin induced maximal aggregation after 5 min at 37°C and pre‐treatment with 10 μM indometacin, 10 μM cangrelor or the dual inhibition for 15 min at RT; plotted: Mea*n* ± SD; *n* ≥ 4; statistics: Mixed‐effects analysis, *p < 0.05, **p < 0.01.

Previously, it was shown that hemin is a strong activator of platelets and platelet‐dependent thrombus formation [9, 10]. Dual inhibition of platelet function via P2Y_12_ and COX‐1 blockers did not show to attenuate hemin‐induced platelet aggregation [10]. However, in our current study we found that at low concentrations of hemin (3.1 and 6.25 μM) pretreatment of isolated human platelets with cangrelor (P2Y_12_‐inhibition), indometacin (COX‐1‐inhibition), or both (dual inhibition: cangrelor + indometacin) significantly reduced the hemin‐induced aggregation response (6.25 μM hemin vs. 10 μM cangrelor, 10 μM indometacin or dual inhibition + 6.25 μM hemin: *p* < 0.01) (Figure 1A,C). However, no substantial inhibition in the presence of platelet antagonists has been seen when platelets were stimulated with high hemin concentrations (12.5 and 25 μM) (Figure 1B,C). Only recently we described that high but not low concentrations of hemin results in plasma membrane destruction, ferroptosis‐associated cell death, and microvesicle formation [8, 15]. This indicates that hemin‐induced platelet ferroptosis which primarily or exclusively requires high hemin concentrations is not regulated by classical signaling pathways required for platelet activation. Ferroptosis is a programmed cell death dependent on iron overload characterized by formation of lipid peroxides and destruction of the plasma membrane which is well described in nucleated cells [19].

### 3.2 Inhibition of Receptor‐Dependent ITAM‐Signaling Inhibits Hemin‐Induced Platelet Activation but Not Plasma Membrane Disintegration

**FIGURE 2:**
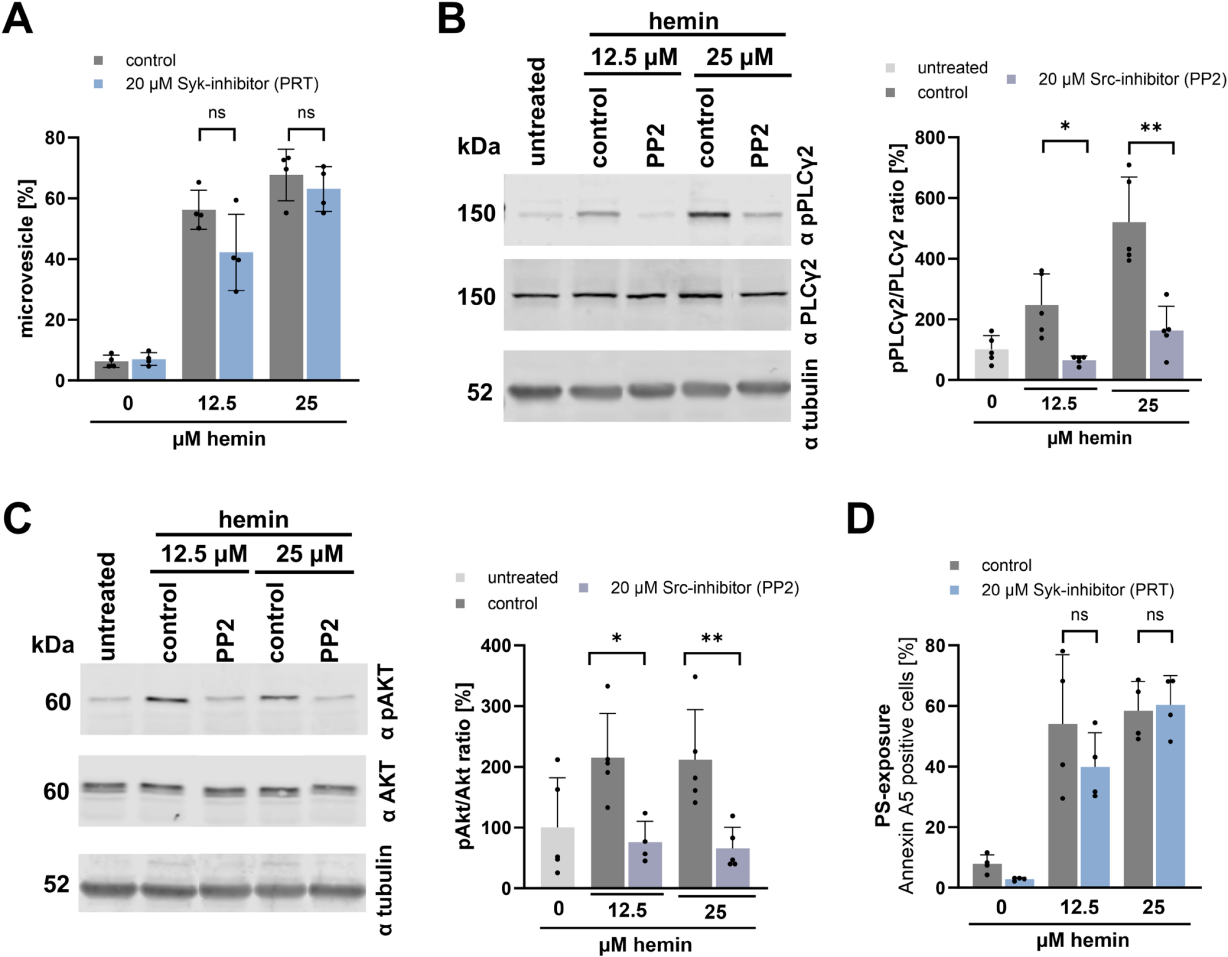
Effect of receptor‐dependent ITAM‐signaling on hemin‐induced platelet activation and plasma membrane disintegration. (A) Flow cytometry measurement of microvesicles (< 1 μm) after 30 min of incubation with hemin and preincubation with 20 μM Syk‐inhibitor (PRT‐060318) for 5 min at RT; plotted: Mean ± SD; *n* = 4; statistics: Wilcoxon matched‐pairs signed rank test, ns not significant. (B) Left: Representative immunoblot image presenting the 12.5 and 25 μM hemin‐induced PLCγ2 phosphorylation (Tyr759) on isolated platelets with pre‐incubation of 20 μM PP2 for 5 min at RT. Right: statistical analysis; plotted: Mean ± SD; *n* = 5; statistics: Student's paired t‐test, *p < 0.05, **p < 0.01. (C) Left: Representative immunoblot image presenting the 12.5 and 25 μM hemin‐induced Akt phosphorylation (Ser473) on isolated platelets with pre‐incubation of 20 μM PP2 for 5 min at RT. Right: statistical analysis; plotted: Mea*n* ± SD; *n* ≥ 4; statistics: Student's paired *t*‐test, *p < 0.05, **p < 0.01. (D) Flow cytometry measurement of phosphatidylserine exposure (PS) after 30 min incubation with hemin on platelets measured with Annexin A5:: FITC (AnxA5); plotted: Mean ± SD; *n* = 4; statistics: Wilcoxon matched‐pairs signed rank test, ns not significant.

Stimulation of CLEC‐2 and GPVI leads to immunoreceptor‐tyrosine‐based‐activation‐motif (ITAM) receptor‐based pathway activation of Syk and PLCγ2 [9, 10, 17]. Thus, we asked whether inhibition of ITAM signaling impacts hemin‐induced plasma membrane disintegration. As shown previously [8, 15] and further substantiated in the present study, high concentrations of hemin induce fragmentation and microvesicle formation (Figure 2A). In the presence of a Syk‐inhibitor (PRT‐060318), hemin‐dependent microvesicle formation could not be attenuated at high concentrations (12.5 and 25 μM) (Figure 2A). However, phosphorylation of PLCγ2 and Akt was significantly decreased by inhibition of ITAM signaling (PP2) (Figure 2B,C). Further, phosphatidylserine exposure (Annexin 5A binding) was not altered by ITAM‐inhibition (PRT‐060318) in the presence of high hemin concentrations (Figure 2D). This indicates that ITAM‐signaling is involved in hemin‐dependent platelet activation but does not play a pivotal role in hemin‐associated plasma membrane disintegration.

### 3.3 Blocking the GPVI Receptor With Anti‐GPVI Inhibits Platelet Activation but Not Plasma Membrane Disintegration

**FIGURE 3:**
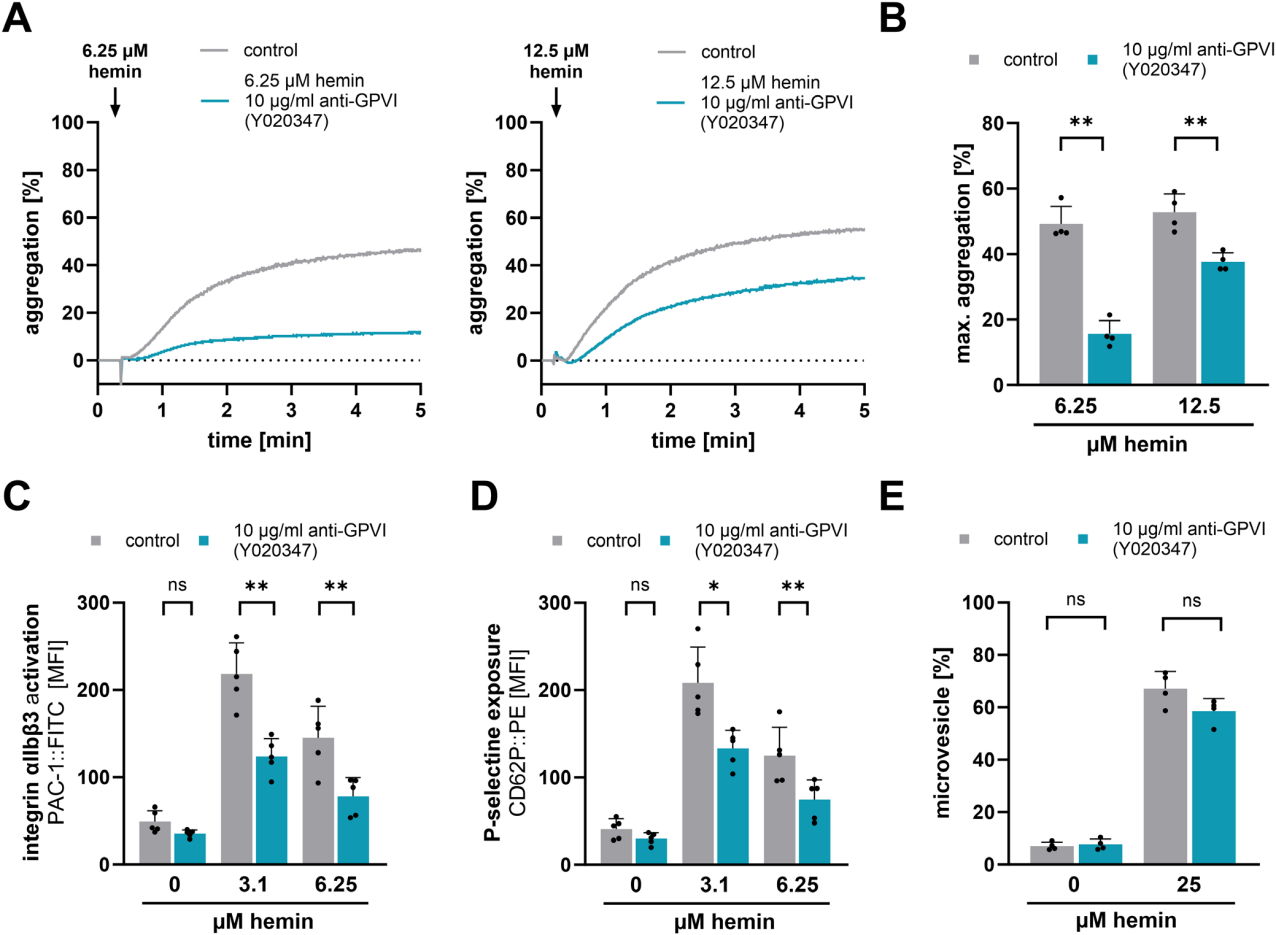
Anti‐GPVI effects on hemin‐induced platelet aggregation, activation, degranulation and microvesicle formation. (A, B) Light transmission aggregometry measurements. (A) Representative traces of platelet aggregation induced with 6.25 and 12.5 μM hemin and 15 min pre‐treatment of 10 μg/mL anti‐GPVI (Fab Y020347) at RT. (B) Hemin‐induced maximal platelet aggregation after 5 min at 37°C and pre‐treatment with 10 μg/mL anti‐GPVI; plotted: Mean ± SD; *n* = 4; statistics: Student's paired *t*‐test, **p < 0.01. (C, D) Flow cytometry measurements of (C) PAC‐1::FITC and (D) CD62P::PE induced by hemin for 30 min at RT and if indicated pre‐treatment with 10 μg/mL anti‐GPVI: plotted: Mean ± SD; *n* = 5; statistics: Student's paired *t*‐test, *p < 0.05, **p < 0.01, ns, not significant. (E) Flow cytometry measurements of microvesicle (< 1 μm) and pre‐incubated with 10 μg/mL anti‐GPVI (Y020347) for 15 min at RT; plotted: Mean ± SD; *n* = 4; statistics: Student's paired *t*‐test, ns not significant.

To further strengthen the findings that the ITAM‐signaling does not have a pivotal influence on plasma membrane rupture, a specific antibody fragment (Fab Y020347) against the GPVI receptor on platelets was used to block the extracellular receptor‐dependent signaling pathway of hemin (Figure 3). Isolated human platelets were preincubated with the blocking antibody (10 μg/mL anti‐GPVI) and activated with hemin. 12.5 and 25 μM hemin‐induced platelet aggregation was significantly inhibited by anti‐GPVI (12.5 μM hemin or 25 μM hemin vs. 10 μg/mL anti‐GPVI + 12.5 μM hemin or 25 μM hemin: *p* < 0.01) (Figure 3A,B). The integrin activation αIIbβ3 and the α‐degranulation were also significantly inhibited by blocking the GPVI receptor (Figure 3C,D). In contrast, the microvesicle formation could not be inhibited by an anti‐GPVI Fab fragment (Figure 3E). This indicates that the microvesicle formation occurs primally via a receptor‐independent signaling pathway of hemin.

### 3.4 Deferoxamine Prevents Hemin‐Induced Platelet Membrane Disintegration and Platelet Death

**FIGURE 4:**
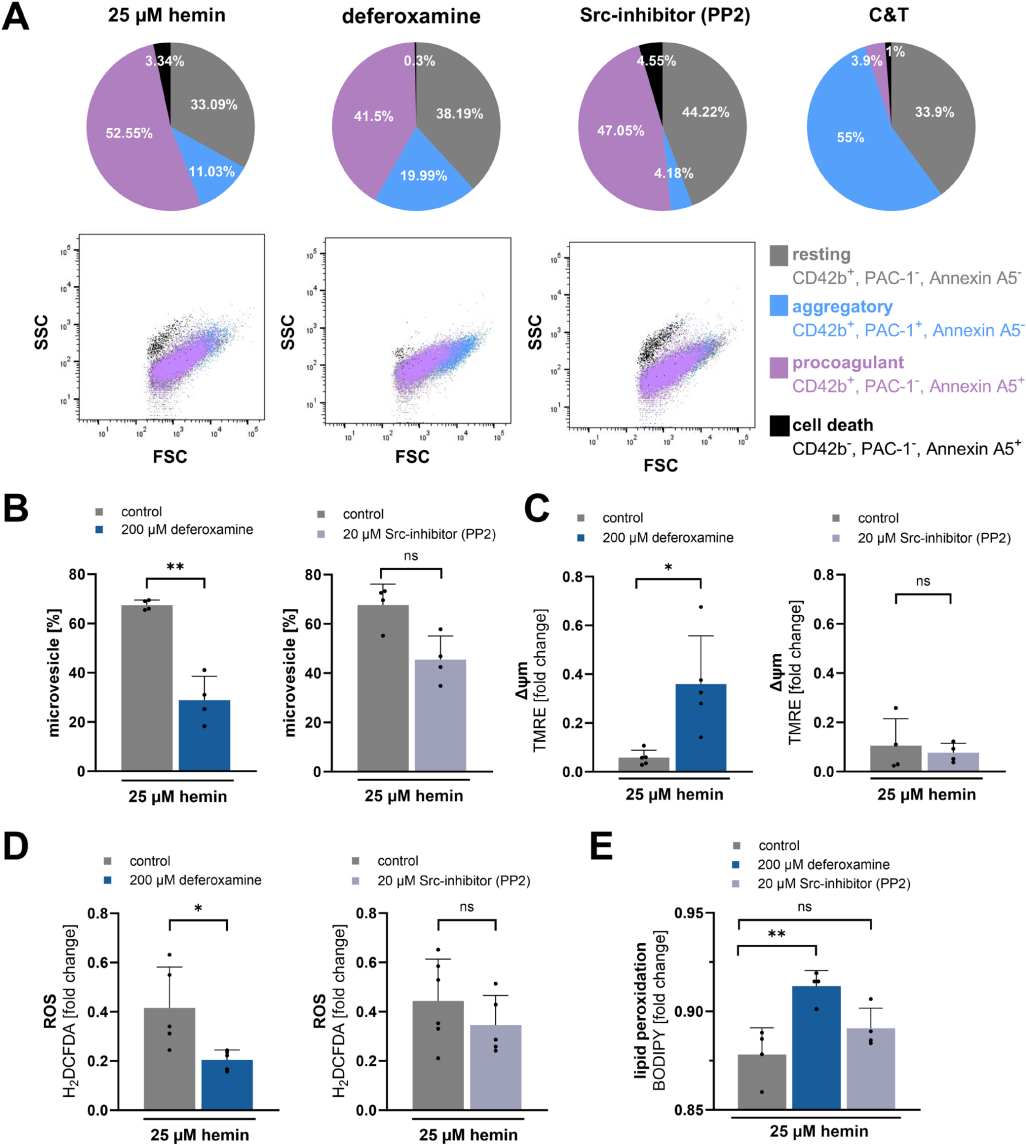
Deferoxamine prevents hemin‐induced platelet membrane disintegration and platelet death. (A) Upper row: Subtype distribution in percent of hemin‐treated isolated human platelets and preincubation with 200 μM deferoxamine for 15 min at RT and 20 μM PP2 for 5 min at RT measured by multi‐color flow cytometry, the 5 μg/mL CRP and 0.1 U/mL Thrombin (C&T) treated samples were used as control (anti‐CD42b::PE, PAC‐1::FITC, Annexin A5::APC); plotted: Mean; *n* = 5. Lower row: representative SSC/FSC dot plots of the four subpopulations (resting, aggregatory, procoagulant, cell death) under each treatment. (B) Flow cytometry measurement of microvesicles (< 1 μm) with beads. Left: preincubation with 200 μM deferoxamine for 15 min at RT; plotted: Mean ± SD; *n* = 4; statistics: Student's paired *t*‐test, **p < 0.01. Right: 20 μM Src‐inhibitor (PP2) for 5 min at RT; plotted: Mean ± SD; *n* = 4; statistics: Student's paired t‐test, ns not significant. (C) Flow cytometry measurement of mitochondrial potential (ΔΨm) with TMRE of hemin‐stimulated platelets. Left: preincubated with 200 μM deferoxamine for 15 min at RT; plotted: Mean ± SD; *n* = 5; statistics: Student's paired t‐test, *p < 0.05. Right: preincubation with 20 μM PP2 for 5 min at RT; plotted: Mean ± SD; *n* = 4; statistics: Student's paired *t*‐test, ns not significant. (D) ROS measurement with H_2_DCFDA of hemin‐treated platelets. Left: preincubation with 200 μM deferoxamine for 15 min at RT; plotted: Mean ± SD; *n* = 5; statistics: Student's paired *t*‐test, *p < 0.05. Right: preincubation with 20 μM PP2 for 5 min at RT; plotted: Mea*n* ± SD; *n* ≥ 5; statistics: Student's paired *t*‐test, ns not significant. (E) Detection of lipid peroxidation with a lipid peroxidation sensor BODIPY C11 after hemin stimulation and pre‐treatment with 200 μM deferoxamine for 15 min at RT or 20 μM PP2 for 5 min at RT; plotted: Mean ± SD; *n* = 4; statistics: RM one‐way ANOVA, **p < 0.01, ns, not significant.

Since inhibition of CLEC‐2/GPVI‐dependent signaling pathways does not substantially attenuate hemin‐induced plasma membrane disintegration, we tested whether direct iron chelation preserves plasma membrane integrity. Hemin is an iron‐containing protoporphyrin and direct integration or uptake of hemin results in iron overload and ferroptotic cell death [20, 26, 27]. Thus, we asked whether the iron chelating compound deferoxamine (DFO) preserves plasma membrane integrity and ferroptotic cell death in platelets upon hemin treatment. We performed multi‐color flow cytometry with the combination of anti‐CD42b and anti‐integrin αIIbβ3 (PAC‐1) antibodies as well as Annexin A5, as described previously [8, 15]. We found that hemin induces in high concentrations (25 μM) a procoagulant population and a population of platelets undergoing cell death (Figure 4A). In comparison, after 30 min of incubation, the combination of CRP and thrombin induces primarily an aggregatory platelet subpopulation (Figure 4A). Interestingly, the formation of procoagulant and cell death platelet subpopulations can only be inhibited by DFO and not by inhibiting the ITAM‐signaling via Src‐inhibitor (PP2) (Figure 4A). Additionally, we observed that the hemin‐dependent microvesicle formation was significantly decreased in the presence of deferoxamine but not by PP2 (25 μM hemin vs. 200 μM deferoxamine + 25 μM hemin: *p* < 0.01) (Figure 4B). Furthermore, metabolic pathways such as mitochondrial membrane depolarization, ROS formation, and lipid peroxidation are known to play a critical role in platelet ferroptosis. The hemin‐induced reduction of mitochondrial membrane potential (ΔΨm) was significantly inhibited by DFO but not by PP2 (25 μM hemin vs. 200 μM deferoxamine + 25 μM hemin: *p* < 0.05) (Figure 4C), and a similar effect was observed on ROS formation (25 μM hemin vs. 200 μM deferoxamine + 25 μM hemin: *p* < 0.05) (Figure 4D). The lipid peroxidation was detected by application of the lipid peroxidation sensor BODIPY‐C11 [28]. With decreasing fluorescence signal, the lipid peroxidation rises and can be significantly mitigated by DFO but not by PP2 (25 μM hemin vs. 200 μM deferoxamine + 25 μM hemin: *p* < 0.01) (Figure 4E). Thus, all ferroptosis markers can be inhibited by deferoxamine but not the hemin‐induced platelet aggregation as well as PLCγ2 and Akt phosphorylation (Figure S2A–D). This indicates that iron chelation mitigates hemin‐dependent platelet membrane disintegration but does not modulate ITAM‐signaling.

### 3.5 Morphological Changes of Hemin‐Stimulated Platelets Are Prevented Through Deferoxamine

**FIGURE 5:**
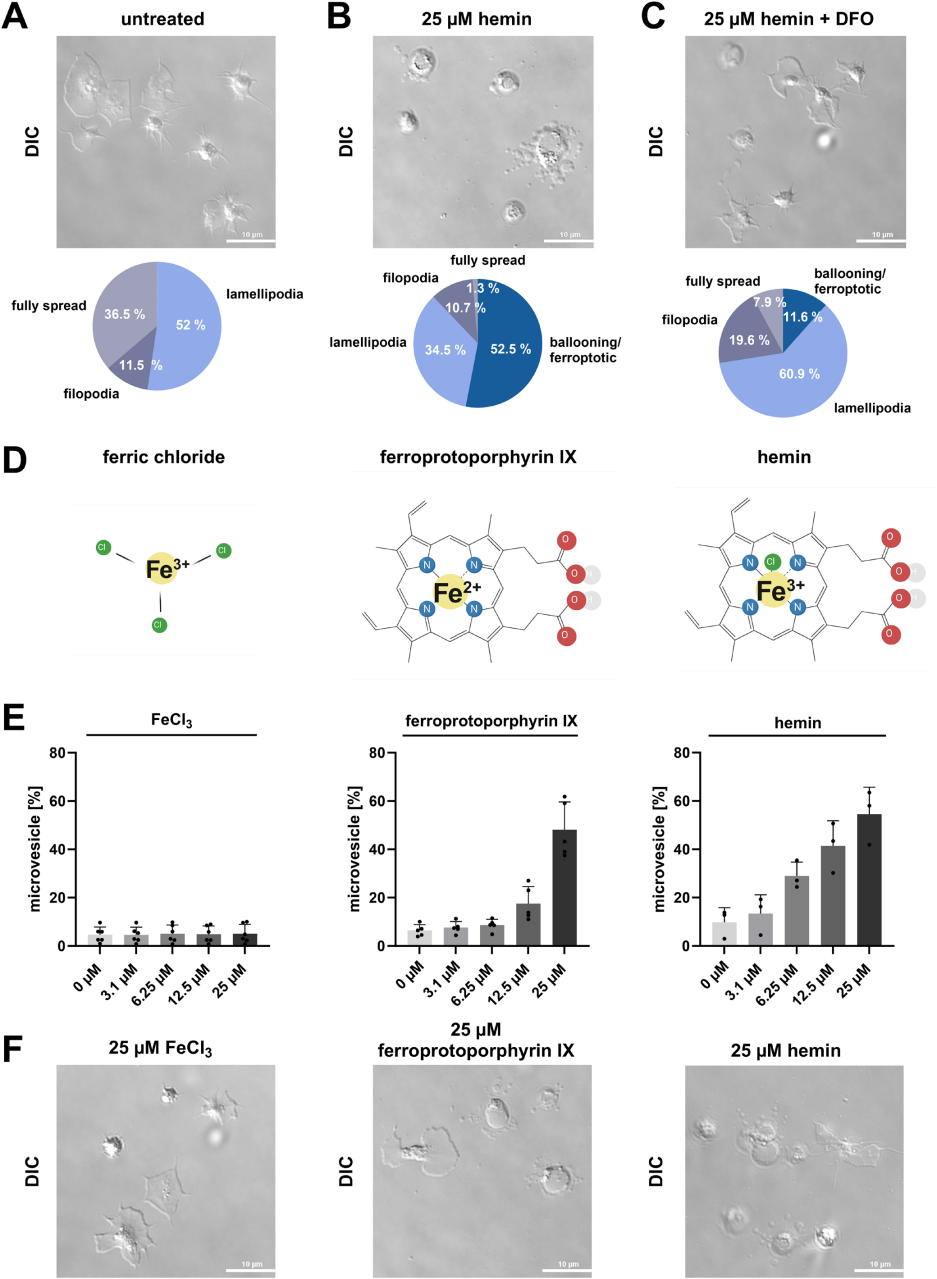
Effect of deferoxamine on hemin‐induced morphological changes and influence of ferric chloride and ferroprotoporphyrin IX on plasma membrane disintegration. (A–C) Live‐cell imaging of platelets spread on 100 μg/mL fibrinogen coated coverslips and activated with (B) 25 μM hemin for 30 min at RT, (C) if indicated pre‐incubation with 200 μM deferoxamine for 15 min at RT; Upper row: DIC images after 30 min incubation with hemin, Lower Row: Representative images of phenotype distribution; Plotted: Mean in % from 3 different healthy donors; scale bar = 10 μm. (D) Chemical structures of ferric chloride, ferroprotoporphyrin IX and hemin (created with Biorender). (E) Flow cytometry measurement of microvesicle generated from isolated human platelets with FeCl_3_, ferroprotoporphyrin IX and hemin after 30 min of incubation at RT. Plotted: Mean ± SD; *n* ≥ 3. (F) Live‐cell imaging of platelets spread on fibrinogen and activated with 25 μM FeCl_3_, ferroprotoporphyrin IX or hemin. Images were taken after 30 min of stimulation. Scale bar = 10 μm.

Recently we demonstrated that hemin‐stimulated platelets spread on fibrinogen undergo morphological changes [15]. Platelets adhering to immobilized fibrinogen fully spread, build lamellipodia or filopodia (Figure 5A). In the presence of high hemin concentrations (25 μM) platelets form primarily a ballooning/ferroptotic phenotype, while the fully spread and lamellipodia phenotype decreases (Figure 5B). At 25 μM hemin induces formation of filopodia, followed by swelling of the platelet, leading to cell rupture. Previously, this type of cell rupture has been associated with ferroptosis and is likely caused by the influx of extracellular ions and water through pores formed in the cell membrane [29]. Deferoxamine is chelating the iron in hemin and inhibits the ferroptotic processes in platelets. Indeed, deferoxamine prevented the formation of ballooning and the ferroptotic phenotype, while promoting the formation of lamellipodia, filopodia and fully spread platelets (ballooning/ferroptotic phenotype: 0% untreated vs. 52.5% 25 μM hemin vs. 11.6% 25 μM hemin + DFO) (Figure 5C). To investigate the role of ferric iron (Fe^3+^) in plasma membrane disruption we compared ferric chloride (Fe^3+^), ferroprotoporphyrin IX (Fe^2+^) and hemin (Figure 5D). Microvesicle formation is a characteristic marker for morphological changes and plasma membrane damage and reflects the receptor‐independent pathway. Therefore, we quantified microvesicle formation after treatment of isolated platelets with FeCl_3_ (ferric iron), ferroprotoporphyrin IX (protoporphyrin ring with ferrous iron) and hemin (protoporphyrin ring with ferric iron) (Figure 5D,E). We observed that FeCl_3_ did not induce microvesicle formation. In contrast, ferroprotoporphyrin IX caused microvesicle formation in a concentration dependent manner, similar to hemin, although with slightly reduced effects (Figure 5E). In addition, we performed live‐cell imaging to assess whether treatment with ferric iron or ferroprotoporphyrin IX results in morphological changes consistent with a ballooning/ferroptotic phenotype (Figure 5F). Notably, Fe^3+^ alone did not induce this phenotype, whereas ferroprotoporphyrin IX, similar to hemin, triggered pronounced ballooning/ferroptotic morphological changes (Figure 5F). These findings indicate that the protoporphyrin ring is essential for the receptor‐independent pathway of hemin‐induced plasma membrane disruption.

### 3.6 Scavenging of Hemin by Soluble GPVI Inhibits Hemin‐Dependent Platelet Activation and Plasma Membrane Disintegration

**FIGURE 6:**
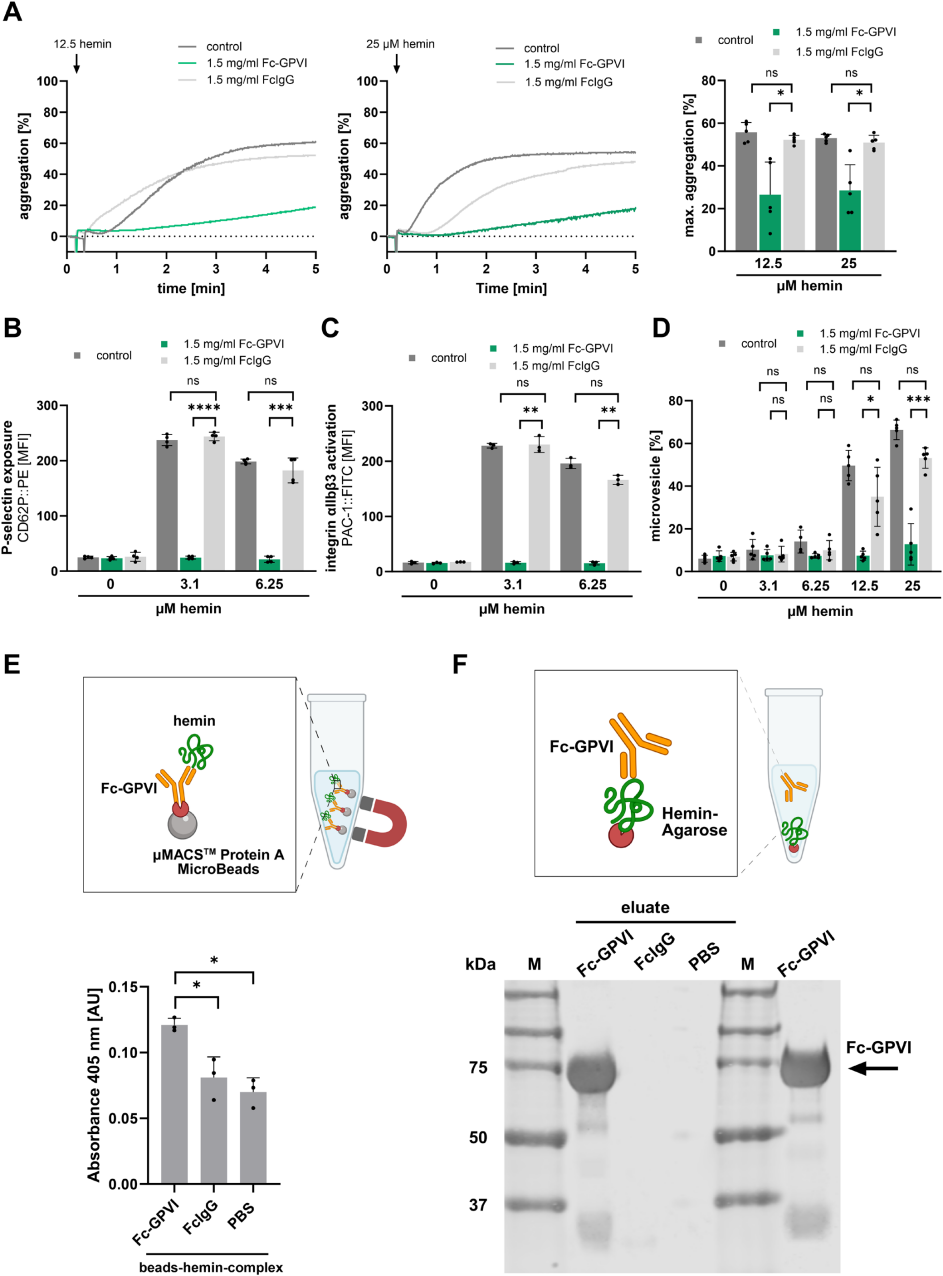
Soluble GPVI inhibits hemin‐induced platelet aggregation, activation, and plasma membrane disintegration. (A) Light transmission aggregometry measurements. Left: Representative traces of 12.5 and 25 μM hemin‐induced aggregation with Fc‐GPVI/hemin‐ or Fc‐IgG/hemin‐mixture. Right: Maximal aggregation induced with the Fc‐GPVI/hemin‐ or Fc‐IgG/hemin‐mixture after 5 min at 37°C; plotted: Mean ± SD; *n* = 5; statistics: RM one way ANOVA, *p < 0.05, ns, not significant. (B, C) Flow cytometry measurements of (B) CD62P::PE and (C) PAC‐1::FITC induced by hemin for 30 min or the Fc‐GPVI/hemin‐ or FcIgG/hemin‐mixture at RT; plotted: Mea*n* ± SD; *n* ≥ 3; statistics: RM one way ANOVA, **p < 0.01, ***p < 0.001, ****p < 0.0001, ns not significant. (D) Flow cytometry measurement of microvesicles (< 1 μm) generated with hemin and the Fc‐GPVI/hemin‐ or Fc‐GPVI/Fc‐IgG‐mixture; plotted: Mea*n* ± SD; *n* ≥ 4; statistics: RM one way ANOVA, *p < 0.05, ***p < 0.001, ns not significant. (E) Incubation of hemin and Fc‐GPVI with superparamagnetic Protein A Beads and separation with a magnet. Absorbance at 405 nm of beads‐hemin‐complex. Plotted: Mean ± SD; *n* = 3; statistics: Student's paired *t*‐test, *p < 0.05. (F) Incubation of Fc‐GPVI, FcIgG or PBS with hemin‐agarose and elution. Immunoblot showing the detection of Fc‐GPVI in the eluates and a Fc‐GPVI loading control (last lane).

Next, we asked whether scavenging of extracellular hemin through a soluble GPVI receptor (Revacept) can prevent platelet activation and plasma membrane disintegration [30]. Hemin was preincubated with a recombinant Fc‐conjugated form of soluble GPVI (Fc‐GPVI) or a Fc‐control fragment [30]. The concentration of Fc‐GPVI was chosen after a titration of Fc‐GPVI with the FcIgG control in light transmission aggregometry experiments (Figure S3A–C). Interestingly, the Fc‐GPVI but not the Fc‐control inhibited hemin‐dependent platelet aggregation (1.5 mg/mL Fc‐GPVI +12.5 μM hemin vs. 1.5 mg/mL FcIgG +12.5 μM hemin: *p* < 0.05) (Figure 6A). Further, degranulation of α‐granules that results in surface receptor expression of P‐selectin and activation of the fibrinogen receptor in the presence of hemin was significantly inhibited by Fc‐GPVI (Figure 6B,C). Further, plasma membrane fragmentation and microvesicle formation of platelets was substantially reduced by scavenging of hemin via soluble GPVI (1.5 mg/mL Fc‐GPVI +12.5 μM hemin vs. 1.5 mg/mL FcIgG +12.5 μM hemin: *p* < 0.05; 1.5 mg/mL Fc‐GPVI +25 μM hemin vs. 1.5 mg/mL FcIgG +25 μM hemin: *p* < 0.001) (Figure 6D). This indicates that soluble GPVI can function as a scavenger receptor to prevent both hemin from interacting with its respective surface receptors, as well as to inhibit hemin uptake and subsequent iron overload, thereby protecting against ferroptosis. First, the direct binding of Fc‐GPVI and hemin was demonstrated by measuring the absorbance of hemin at 405 nm after incubation of Fc‐GPVI loaded beads with hemin (Figure 6E). The resulting Fc‐GPVI‐hemin‐beads complex showed a significantly higher absorbance compared with FcIgG and PBS controls, indicating that hemin binds to Fc‐GPVI (Fc‐GPVI vs. FcIgG: *p* < 0.05) (Figure 6E). To further confirm this interaction, Fc‐GPVI was immunoprecipitated with hemin‐agarose beads (Figure 6F). Indeed, Fc‐GPVI was detected in the eluate of the Fc‐GPVI‐hemin‐agarose mixture but not in the eluates from similarly treated FcIgG and PBS control samples, confirming a strong binding of Fc‐GPVI to hemin (Figure 6F).

## 4 Discussion

The major findings of the present study are: (i) P2Y_12_ and COX‐1 blockers attenuate platelet aggregation stimulated by low hemin concentrations but not high hemin concentrations (ii) ITAM‐signaling inhibition does not attenuate hemin‐induced plasma membrane disintegration (iii) deferoxamine prevents hemin‐induced platelet membrane disintegration and platelet death and (iv) scavenging of hemin through soluble GPVI receptor prevents both platelet activation and ferroptotic plasma membrane disintegration.

Recently, it has been shown that hemin interacts with the glycoprotein VI (GPVI) and C‐type lectin‐like receptor 2 (CLEC‐2), which leads to platelet activation and aggregation [9, 10]. Conventional platelet antagonists, such as P2Y_12_‐antagonists (cangrelor) and COX‐1 inhibitor (indometacin) have not been shown to inhibit platelet aggregation induced by high concentrations of hemin [10]. Our findings show that hemin induces in a concentration‐dependent manner platelet aggregation and support previous observations that platelet aggregation at high hemin concentrations is resistant to dual platelet inhibition. In contrast, we found that at low hemin concentrations platelet aggregation can be inhibited by dual inhibition of P2Y_12_ and COX‐1. These results suggest that at high hemin concentrations, additional signaling pathways beyond the classical P2Y_12_ and COX‐1 dependent mechanisms contribute to platelet activation. Upon platelet degranulation the nucleotide ADP is released and binds as an autocrine messenger back to the P2Y_12_ receptor and thus enhances the platelet activation. The synthesis of thromboxane A_2_ also leads to autocrine effects on platelets and enhances the platelet activation through binding to the TP receptor. Both degranulation and thromboxane A_2_ synthesis rise with increasing hemin concentrations [17]. Therefore, activation with higher concentrations of hemin leads to a stronger amplification of platelet response. Nevertheless, low hemin concentrations can be inhibited by blocking the P2Y_12_ receptor with cangrelor and by inhibiting COX‐1 with indometacin (Figure 1). However, the dual inhibition of hemin‐induced platelet aggregation is ineffective at higher concentrations of hemin (12.5 and 25 μM). It is likely that at high concentrations, receptor inhibition is no longer sufficient and hemin induces platelet plasma membrane disruption in a receptor‐independent manner. As a result, agglutination rather than classical aggregation dominates, which cannot be inhibited by P2Y_12_ or COX‐1 inhibitors. Previously, it was demonstrated that hemin causes cellular iron accumulation and induces a non‐apoptotic form of cell death in nucleated cells, known as ferroptosis [19]. The incorporated ferric iron in hemin can generate reactive oxygen species and cause lipid peroxidation via the Haber‐Weiß reaction and Fenton reaction [31]. Lipid peroxidation is accompanied by a significant change in the plasma membrane, which occurs primarily at high hemin concentrations (> 12.5 μM hemin) [8, 15]. We found that high hemin concentrations (12.5 and 25 μM) induce microvesicle formation, phosphatidylserine exposure, the formation of procoagulant platelets and a cell death associated platelet subpopulation. These effects cannot be diminished by inhibition of the hemin‐induced ITAM signaling, even though inhibition of the ITAM signaling attenuates the hemin‐induced PLCγ2 and Akt phosphorylation. Notably, the microvesicle formation, phosphatidylserine exposure and cell death associated subpopulation can be significantly inhibited by deferoxamine, an iron chelator. Deferoxamine is approved for treating iron overload in thalassemia patients [32]. This indicates that the effects are primarily iron‐dependent but ITAM signaling independent. Moreover, we observed that classical ferroptosis markers such as the generation of ROS, the lipid peroxidation and loss of mitochondrial membrane potential were reduced by the chelation of iron via deferoxamine but not via the inhibition of the ITAM signaling pathway. Thus, we conclude that high hemin concentrations (12.5 and 25 μM) lead to plasma membrane disintegration and ferroptosis in platelets (iron overload), mediated by the iron incorporated in hemin but independent of ITAM‐receptor signaling. Notably, Fe^3+^ did not induce plasma membrane disintegration, whereas ferroprotoporphyrin IX, similar to hemin, triggered pronounced ballooning/ferroptotic morphological changes. These findings indicate that the protoporphyrin ring is essential for the receptor‐independent pathway of hemin‐induced plasma membrane disruption. We propose that the hydrophobic hemin intercalates into and diffuses through the platelet plasma membrane, thereby shuttling the iron into the cell and allowing intracellular iron‐dependent ferroptotic processes to occur. In contrast, free ferric iron cannot diffuse through the cell membrane and thus would require active uptake by platelets. Therefore, Fe^3+^ does not induce membrane damage or microvesicle release. In addition, Tsukiji et al. demonstrated that ferric iron activates platelets via the CLEC‐2 receptor, leading to platelet activation and aggregation [33]. These findings indicate that free ferric iron can stimulate platelet signaling through ITAM‐dependent pathways but does not cause receptor‐independent membrane disruption. Our findings confirm that hemin at high concentrations results in receptor‐independent plasma membrane damage mediated by the protoporphyrin structure and intracellular iron‐dependent mechanisms.

Further, this study complements previous studies and extends our understanding of the hemin‐induced aggregation, activation, and plasma membrane disintegration. The previously published data focused only on one concentration, and the connection between the ITAM receptor pathway and the receptor‐independent pathway was not directly addressed. Bourne et al. [10] and Oishi et al. [9] demonstrated a concentration‐dependent hemin‐induced aggregation and activation, as well as concentration‐dependent phosphatidylserine exposure. However, they showed only with one concentration of 6.25 μM hemin that Src‐inhibition (PP2) diminishes the hemin‐induced aggregation; whether at higher hemin concentrations the effect of inhibition of ITAM signaling remains unclear. NaveenKumar et al. [20] focused on platelet ferroptosis and described the hemin‐induced ferroptosis in platelets with increased ROS levels and lipid peroxidation. The platelets were stimulated with concentrations between 0 and 25 μM hemin. Nevertheless, inhibition studies with the iron‐chelator deferoxamine were done with only 10 μM of hemin. Without including the other hemin concentrations, the conclusions about inhibition of ferroptosis are limited. Furthermore, NaveenKumar et al. measured only CD62P as an activation marker, which was again performed with one single hemin concentration. In order to bridge the gap between receptor activation pathway and ferroptosis, we compared high hemin concentrations (12.5 and 25 μM) across aggregation assays, activation experiments, and ferroptosis marker analyses with ITAM receptor inhibitors and deferoxamine, an iron‐chelator, to distinguish between receptor‐dependent and independent pathways. We were able to demonstrate that high concentrations of hemin induce both ITAM receptor–dependent platelet activation and ferroptosis. However, only the ferroptotic markers were inhibited by deferoxamine, whereas receptor‐specific activation markers were unaffected (Table S1). Conversely, inhibition of receptor‐specific kinases suppressed only the receptor‐dependent signaling pathway without affecting the receptor‐independent pathway (Table S1).

Furthermore, we observed that hemin is scavenged by a soluble GPVI receptor resulting in significant inhibition of platelet activation, aggregation, and microvesicle formation. These findings demonstrate that hemin is a direct interaction partner of the GPVI receptor and that both the thrombotic and ferroptotic potential of hemin can be effectively suppressed. This understanding is particularly relevant for patients with active bleeding. In trauma patients with ongoing hemorrhage, a reduction of GPVI expression on platelets has been reported, which correlates with decreased platelet responsiveness during bleeding [34]. The released hemin may be responsible for this effect, as Fink et al. showed that hemin induces pronounced GPVI shedding on platelets and an increase in soluble GPVI [17]. This regulatory mechanism could potentially be supported by the administration of a soluble GPVI, allowing the neutralization of hemin during bleeding episodes. This would not only limit further hemolysis and bleeding but also inhibit hemin‐induced platelet activation and aggregation. Harm et al. also demonstrated that patients with chronic coronary syndrome undergoing percutaneous coronary intervention who were treated with a soluble recombinant GPVI experienced fewer adverse bleeding events and that platelet function was more impaired compared to patients receiving a placebo [35].

In summary, our results provide evidence that hemin induces a dual effect on platelet activation, depending on the concentration of extracellular hemin. High hemin concentrations lead to enhanced platelet‐dependent thrombosis and ferroptotic plasma membrane disruption which cannot be prevented by conventional antiplatelet drugs or by the interference of the GPVI/CLEC‐2‐dependent ITAM signaling. We provide a therapeutic strategy via iron‐chelation and/or hemin scavenging through soluble GPVI that might be of benefit at the site of microhemorrhages that play an important pathophysiological role in vulnerable plaques and aneurysm formation.

## Author Contributions

Experiment design and planning: Z.L.; M.P.G.; R.H.; A.‐K.R.; experiment performance: Z.L.; R.H.; data analysis and plotting: Z.L.; R.H.; interpretation and discussion of results: Z.L.; M.P.G.; R.H.; A.‐K.R.; T.C.; P.W.S.; figure design: Z.L.; M.P.G.; manuscript writing: M.P.G., Z.L., A.‐K.R., T.C.

## Funding

This work was supported by the German Research Foundation (DFG)—Project number 335549539—GRK 2381 and from the Reinhard Frank‐Stiftung at the University of Tübingen. R.H. was supported by a research grant from the German Cardiac Society (DGK).

## Conflicts of Interest

Meinrad Paul Gawaz is co‐founder of AdvanceCOR GmbH, the manufacturer of Revacept.

## Supporting information


**Table S1:** Summary of the results. Green: effect; red: no effect. (**↓**) decreased; (**↑**) increased; (−) no effect.
**Figure S1:** Platelet ferroptosis induced by hemin is concentration‐dependent. (A) Measurement of lipid peroxidation with a lipid peroxidation sensor BODIPY C11 after 30 min hemin stimulation at RT; plotted: Mean ± SD; *n* = 5; statistics: RM one‐way ANOVA against black arrow (↓), **p < 0.01, ***p < 0.001, ns not significant. (B) Flow cytometry measurements of phosphatidyl serine (PS) exposure on platelets after 30 min hemin stimulation at RT; Plotted: Mean ± SD; *n* = 5; statistics: RM one‐way ANOVA against black arrow (**↓**), **p < 0.01, ***p < 0.001, ns not significant. (C) Flow cytometry measurements of microvesicles (< 1 μm) after 30 min hemin stimulation at RT; Plotted: Mean ± SD; *n* = 4; statistics: RM one‐way ANOVA against black arrow (**↓**), **p < 0.01, ns not significant. (D) Flow cytometry measurement of mitochondrial potential (△Ψm) with TMRE after 30 min hemin stimulation at RT; Plotted: Mean ± SD; *n* = 5; statistics: RM one‐way ANOVA against black arrow (**↓**), *p < 0.05, ns not significant.
**Figure S2:** Role of deferoxamine in modulating hemin‐induced ITAM‐signaling pathway. (A/B) Light transmission aggregometry measurements. (A) Representative traces of platelet aggregation induced with 12.5 and 25 μM hemin and 15 min pre‐treatment 200 μM deferoxamine at RT. (B) Hemin‐induced maximal platelet aggregation after 5 min at 37°C and pre‐treatment with 200 μM deferoxamine; plotted: Mean ± SD; *n* = 5. (C) Left: Representative immunoblot image presenting the 12.5 and 25 μM hemin‐induced PLCy2 phosphorylation (Tyr759) in isolated human platelets with pre‐incubation of 20 μM PP2 for 5 min at RT and 200 μM deferoxamine for 15 min at RT. Right: statistical analysis; plotted: Mean ± SD; *n* = 4: statistics: RM one‐way ANOVA, *p < 0.05, **p < 0.01. (D) Left: Representative immunoblot image presenting the 12.5 and 25 μM hemin‐induced Akt phosphorylation (Ser473) in isolated human platelets with pre‐incubation of 20 μM PP2 for 5 min at RT and 200 μM deferoxamine for 15 min at RT. Right: statistical analysis; plotted: Mea*n* ± SD; *n* ≥ 4: statistics: Mixed‐effects analysis, *p < 0.05, **p < 0.01.
**Figure S3:** Light transmission aggregometry measurements performed with isolated human platelets, pre‐incubated with different concentrations of soluble GPVI (sGPVI) for 15 min at RT and activated with (A) 5 μg/mL CRP‐A (B) 12.5 μM hemin and (C) 25 μM hemin. Statistical analysis: Plotted area under curve of platelet aggregation after 5 min at 37°C; Plotted: Mea*n* ± SD; *n* ≥ 4; statistics: RM one way ANOVA; *p < 0.05, **p < 0.01, ***p < 0.001.

## Data Availability

For original data, please contact meinrad.gawaz@med.uni-tuebingen.de.
