# Supplementary material for: Receptor‐Dependent and ‐Independent Effects of Hemin on Platelet Plasma Membrane Disintegration
Source: FASEB J. 2026 Jan 12;40(2):e71463. doi: 10.1096/fj.202503706R (PMC12794464; doi:10.1096/fj.202503706R)
Supplement: Supplementary file 1 — Table S1: Summary of the results. Green: effect; red: no effect. (↓) decreased; (↑) increased; (−) no effect. Figure S1: Platelet ferroptosis induced by hemin is concentration‐dependent. (A) Measurement of lipid peroxidation with a lipid peroxidation sensor BODIPY C11 after 30 min hemin stimulation at RT; plotted: Mean ± SD; n = 5; statistics: RM one‐way ANOVA against black arrow (↓), **p < 0.01, ***p < 0.001, ns not significant. (B) Flow cytometry measurements of phosphatidyl serine (PS) exposure on platelets after 30 min hemin stimulation at RT; Plotted: Mean ± SD; n = 5; statistics: RM one‐way ANOVA against black arrow (↓), **p < 0.01, ***p < 0.001, ns not significant. (C) Flow cytometry measurements of microvesicles (< 1 μm) after 30 min hemin stimulation at RT; Plotted: Mean ± SD; n = 4; statistics: RM one‐way ANOVA against black arrow (↓), **p < 0.01, ns not significant. (D) Flow cytometry measurement of mitochondrial potential (△Ψm) with TMRE after 30 min hemin stimulation at RT; Plotted: Mean ± SD; n = 5; statistics: RM one‐way ANOVA against black arrow (↓), *p < 0.05, ns not significant. Figure S2: Role of deferoxamine in modulating hemin‐induced ITAM‐signaling pathway. (A/B) Light transmission aggregometry measurements. (A) Representative traces of platelet aggregation induced with 12.5 and 25 μM hemin and 15 min pre‐treatment 200 μM deferoxamine at RT. (B) Hemin‐induced maximal platelet aggregation after 5 min at 37°C and pre‐treatment with 200 μM deferoxamine; plotted: Mean ± SD; n = 5. (C) Left: Representative immunoblot image presenting the 12.5 and 25 μM hemin‐induced PLCy2 phosphorylation (Tyr759) in isolated human platelets with pre‐incubation of 20 μM PP2 for 5 min at RT and 200 μM deferoxamine for 15 min at RT. Right: statistical analysis; plotted: Mean ± SD; n = 4: statistics: RM one‐way ANOVA, *p < 0.05, **p < 0.01. (D) Left: Representative immunoblot image presenting the 12.5 and 25 μM hemin‐induced Akt phosphorylation (Ser473) in isolated huma [file FSB2-40-e71463-s001.docx]

**Supplemental Material**

**Receptor-dependent and -independent effects of hemin on platelet plasma membrane disintegration**

Zoi Laspa, Anne-Katrin Rohlfing, Ravi Hochuli, Pamela Weronika Sowa, Tatsiana Castor, and Meinrad Paul Gawaz

Department of Cardiology and Angiology, University Hospital Tübingen, University Tübingen, Otfried-Müller-Str. 10, 72076 Tübingen, Germany

**Corresponding author**

Meinrad P. Gawaz, MD

Department of Cardiology and Angiology; University Hospital Tübingen; Eberhard Karls University Tübingen

Otfried-Müller-Str. 10; 72076 Tübingen, Germany

Tel.: +49 7071 29 83688; Fax: +49 7071 29 5749

E-Mail: [meinrad.gawaz@med.uni-tuebingen.de](mailto:meinrad.gawaz@med.uni-tuebingen.de)

**Supplemental Table 1.** Summary of the results. green: effect; red: no effect. (**🠛**) decreased; (**⭡**) increased; (-) no effect.

| **Method** | **Receptor-dependent hemin signaling**  (Src inhibition with PP2) | **Receptor-independent hemin signaling**  (iron chelation with deferoxamine) |
| --- | --- | --- |
| **Hemin induces** | **25 µM hemin** | **25 µM hemin** |
| **platelet aggregation** | **🠛** | **-** |
| **PLCγ2 phosphorylation** | **🠛** | **-** |
| **Akt phosphorylation** | **🠛** | **-** |
| **platelet subpopulations** |  | |
| resting | **⭡** | **-** |
| aggregatory | 🠛 | **⭡** |
| procoagulant | **-** | **🠛** |
| cell death | **-** | **🠛** |
| **microvesicle formation** | **-** | **🠛** |
| **phosphatidylserine exposure** | **-** | **🠛** |
| **mitochondrial potential** | **-** | **🠛** |
| **ROS generation** | **-** | **🠛** |
| **lipid peroxidation** | **-** | **🠛** |

**Supplemental Figure 1**


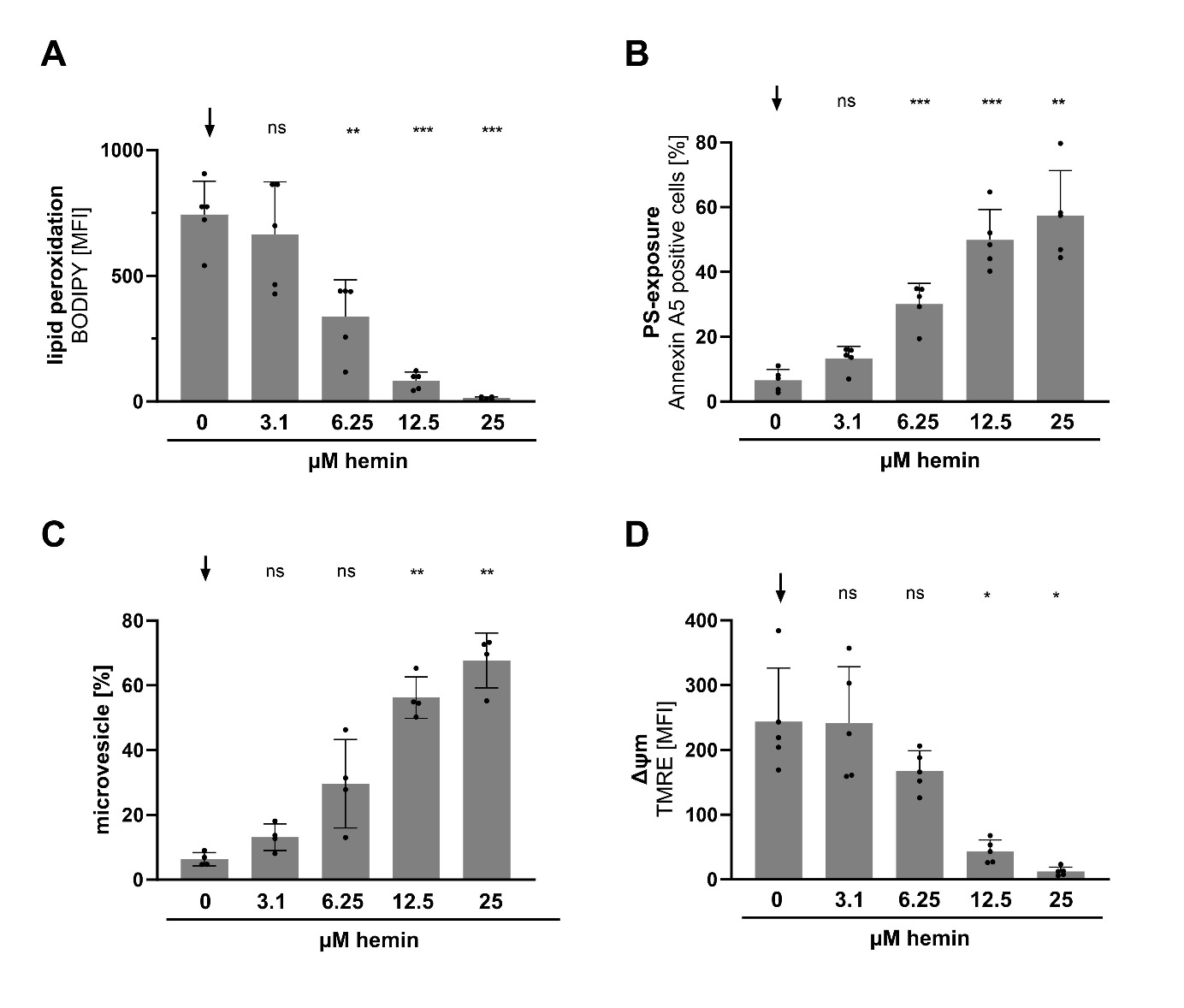


**Supplemental Figure 1. Platelet ferroptosis induced by hemin is concentration-dependent. A** Measurement of lipid peroxidation with a lipid peroxidation sensor BODIPY C11 after 30 min hemin stimulation at RT; plotted: Mean ± SD; n = 5; statistics: RM one-way ANOVA against black arrow (🠛), **p < 0.01, ***p< 0.001, ns not significant. **B** Flow cytometry measurements of phosphatidyl serine (PS) exposure on platelets after 30 min hemin stimulation at RT; Plotted: Mean ± SD; n = 5; statistics: RM one-way ANOVA against black arrow (🠛), **p < 0.01, ***p< 0.001, ns not significant. **C** Flow cytometry measurements of microvesicles (< 1µm) after 30 min hemin stimulation at RT; Plotted: Mean ± SD; n = 4; statistics: RM one-way ANOVA against black arrow (🠛), **p < 0.01, ns not significant. **D** Flow cytometry measurement of mitochondrial potential (ΔΨm) with TMRE after 30 min hemin stimulation at RT; Plotted: Mean ± SD; n = 5; statistics: RM one-way ANOVA against black arrow (🠛), *p < 0.05, ns not significant.

**Supplemental Figure 2**


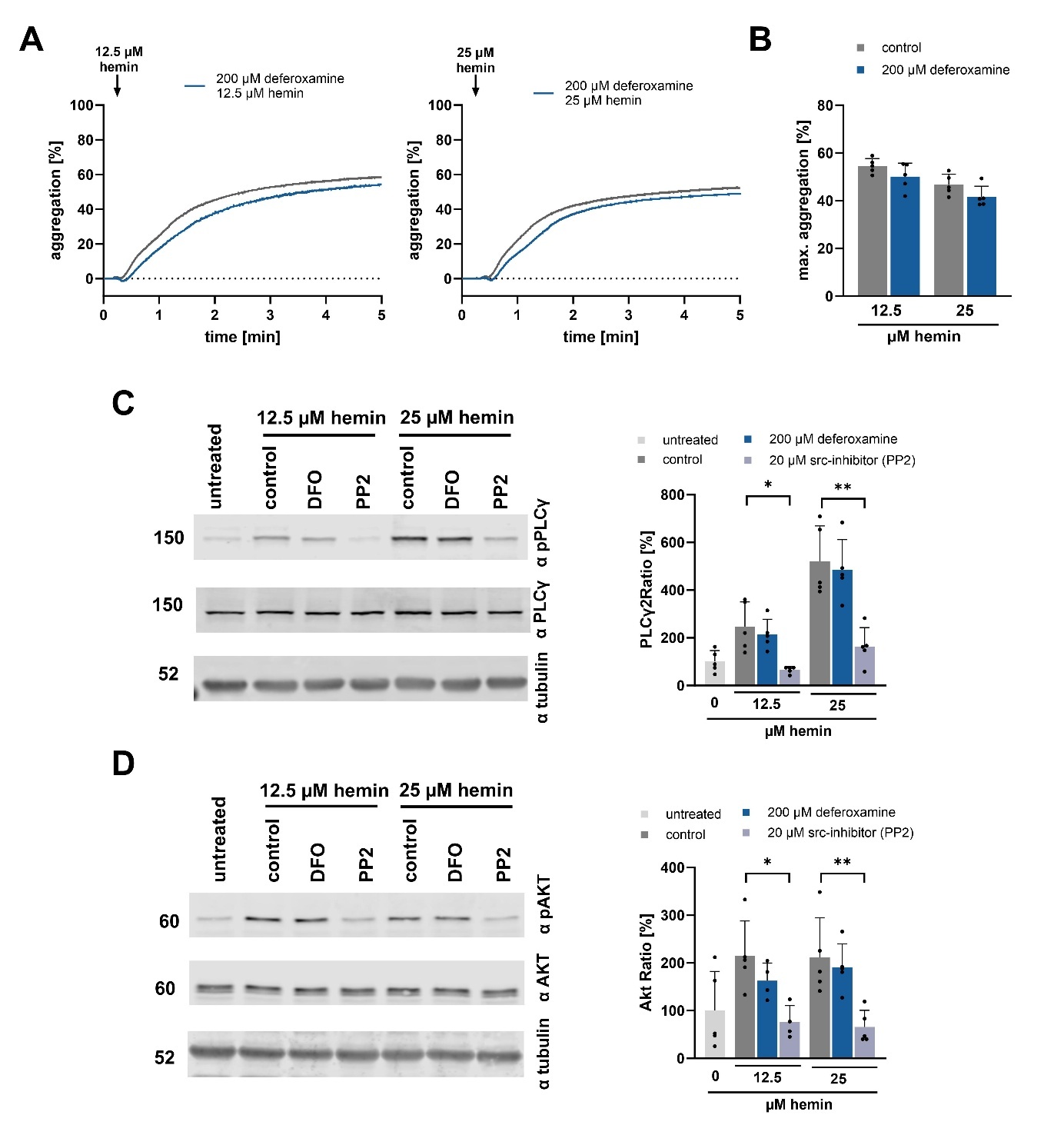


**Supplemental Figure 2. Role of deferoxamine in modulating hemin-induced ITAM-signaling pathway.** (A/B) Light transmission aggregometry measurements. (A) Representative traces of platelet aggregation induced with 12.5 and 25 µM hemin and 15 min pre-treatment 200 µM deferoxamine at RT. (B) Hemin-induced maximal platelet aggregation after 5 min at 37°C and pre-treatment with 200 µM deferoxamine; plotted: Mean ± SD; n =5. (C) Left: Representative immunoblot image presenting the 12.5 and 25 µM hemin-induced PLCy2 phosphorylation (Tyr759) in isolated human platelets with pre-incubation of 20 µM PP2 for 5 min at RT and 200 µM deferoxamine for 15 min at RT. Right: statistical analysis; plotted: Mean ± SD; n = 4: statistics: RM one-way ANOVA, *p < 0.05, **p < 0.01. (D) Left: Representative immunoblot image presenting the 12.5 and 25 µM hemin-induced Akt phosphorylation (Ser473) in isolated human platelets with pre-incubation of 20 µM PP2 for 5 min at RT and 200 µM deferoxamine for 15 min at RT. Right: statistical analysis; plotted: Mean ± SD; n ≥ 4: statistics: Mixed-effects analysis, *p < 0.05, **p < 0.01.

**Supplemental Figure 3**


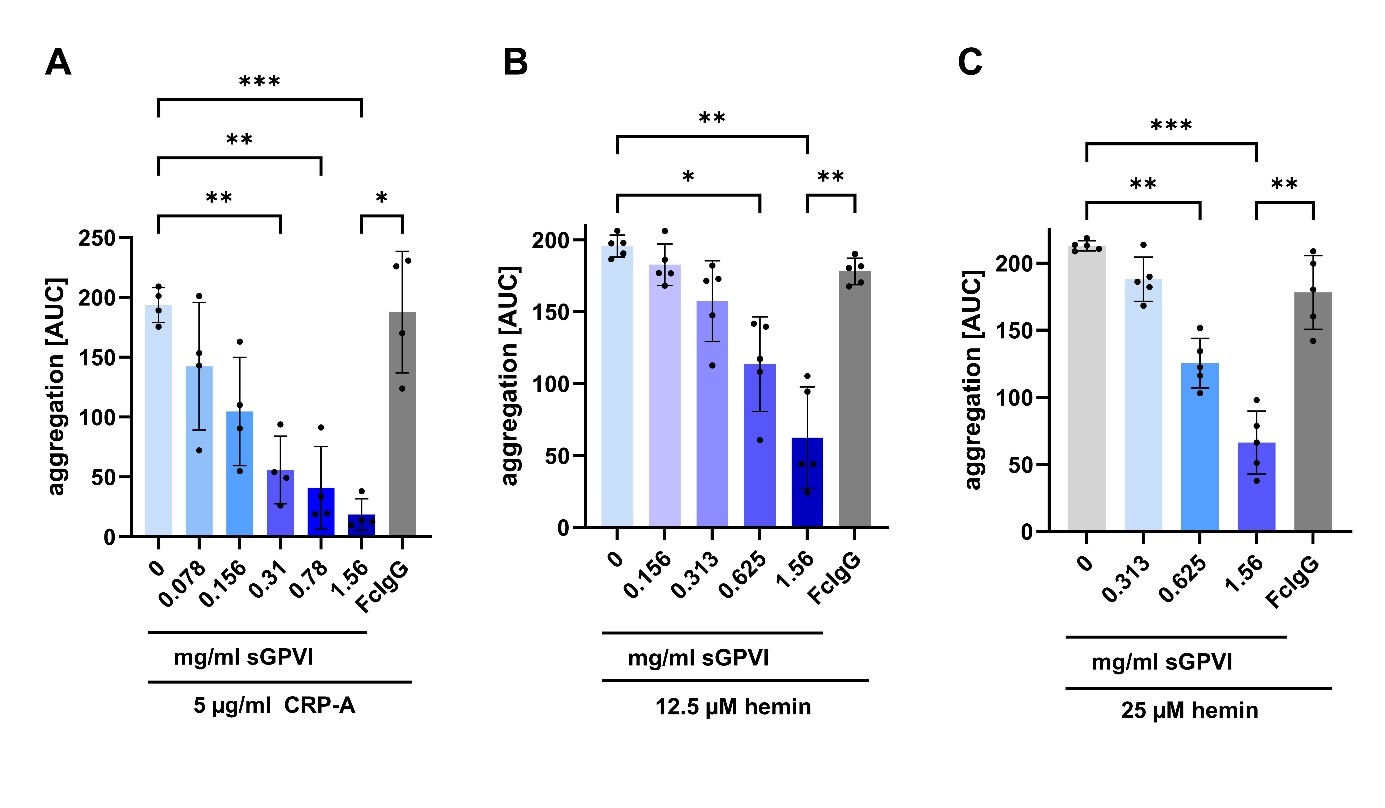


**Supplemental Figure 3.** Light transmission aggregometry measurements performed with isolated human platelets, pre-incubated with different concentrations of soluble GPVI (sGPVI) for 15 min at RT and activated with **A** 5 µg/ml CRP-A **B** 12.5 µM hemin and **C** 25 µM hemin. Statistical analysis: Plotted area under curve of platelet aggregation after 5 min at 37°C; Plotted: Mean ± SD; n ≥ 4; statistics: RM one way ANOVA; *p < 0.05, **p < 0.01, ***p < 0.001.
